# MRI evaluation prior to Transcatheter Aortic Valve Implantation (TAVI): When to acquire and how to interpret

**DOI:** 10.1007/s13244-016-0470-0

**Published:** 2016-02-25

**Authors:** Abhishek Chaturvedi, Susan K. Hobbs, Fred S. Ling, Apeksha Chaturvedi, Peter Knight

**Affiliations:** Department of Imaging Sciences, University of Rochester Medical Center, 601 Elmwood Ave, P.O. Box no. 648, Rochester, NY 14642 USA; Department of Medicine, Cardiology, University of Rochester Medical Center, Rochester, NY USA; Department of Surgery, Cardiac, University of Rochester Medical Center, Rochester, NY USA

**Keywords:** Transcatheter aortic valve implantation, Transcatheter aortic valve replacement, Magnetic resonance imaging, Severe aortic stenosis, Non-contrast MRI

## Abstract

Transcatheter Aortic Valve Implantation (TAVI) is increasingly being used in patients with severe aortic stenosis who are not candidates for surgery. ECG-gated CT angiography (CTA) plays an important role in the preoperative planning for these devices. As the number of patients undergoing these procedures increases, a subset of patients is being recognized who have contraindications to iodinated contrast medium, either due to a prior severe allergic type reaction or poor renal function. Another subgroup of patients with low flow and low gradient aortic stenosis is being recognized that are usually assessed for severity of aortic stenosis by stress echocardiography. There are contraindications to stress echocardiography and some of these patients may not be able to undergo this test. Non-contrast MRI can be a useful emerging modality for evaluating these patients. In this article, we discuss the emerging indications of non-contrast MRI in preoperative assessment for TAVI and describe the commonly used MRI sequences. A comparison of the most important measurements obtained for TAVI assessment on CTA and MRI from same subjects is included.

*Teaching Points*

• *MRI can be used for preoperative assessment of aortic annulus.*

• *MRI is an alternate to CTA when iodinated contrast is contraindicated.*

• *Measurements obtained by non-contrast MRI are similar to contrast enhanced CTA.*

• *MRI can be used to assess severity of aortic stenosis.*

**Electronic supplementary material:**

The online version of this article (doi:10.1007/s13244-016-0470-0) contains supplementary material, which is available to authorized users.

## Background

Valvular heart disease is estimated to account for as many as 20 % of cardiac surgical procedures performed in the United States [1]. Transcatheter aortic valve implantation (TAVI) has become an increasingly promising treatment for high surgical risk patients with severe aortic stenosis. TAVI has resulted in substantially reduced mortality and morbidity compared to standard treatment methods in the poor surgical risk patient subgroup [2]. It has become a viable option in these patients with increasingly promising short- and mid-term outcomes.

Pre-procedure imaging plays an important role in assessing the anatomy of the aortic annulus, aorta, iliac and femoral arteries in these patients, thereby preventing patient-prosthesis mismatch and determining a suitable vascular approach [3]. Accurate prosthesis-valve matching is necessary not only to reduce the incidence and amount of paravalvular regurgitation (if the prosthetic valve is too small), but also aortic rupture (when prosthetic valve is too large). Analyzing the aorta and ilofemoral arteries with attention to size, tortuosity, and calcification determines the optimal prosthetic valve size and access approach. Echocardiography, ECG-gated CT angiography (CTA), and catheter angiography have been used for pre-operative planning. Increasingly, CTA is playing an important role towards preoperative assessment of aortic annular dimensions and aortic geometry for optimal prosthetic valve size and functions next to echocardiography in aortic root evaluation. Evaluation of the peripheral vasculature is routinely being performed using helical CTA of the abdomen and pelvis. However, iodinated CT contrast media cannot be administered to patients with severely impaired renal function or severe contrast allergic type reaction [4], and non-contrast CT obtained in such cases is limited in its ability to evaluate non-calcified plaques and luminal dimensions in vessels. Echocardiography is of limited utility in the presence of extensive vascular calcifications or when an optimum acoustic window is not obtainable. In such patients, invasive catheter angiography, transesophageal ultrasound [5], invasive intravascular ultrasound, or low dose catheter CT angiography [6] have been suggested as alternatives for pre-procedure assessment. Aortic annulus sizing for TAVI by transesophageal echocardiography (TEE) also avoids the use of iodinated contrast. However, large annular size measured by CTA or MRI but not TTE is predictive of paravalvular leak [7]. Aortic regurgitation is the most frequent post-procedural complication after TAVI. Any aortic regurgitation is linked with increased late mortality [8].

A subset of patients is increasingly being recognized when the gradient suggests less severe stenosis than the calculated valve area. This can be due to a dilated ventricle with low ejection fraction (EF) or small ventricle with normal EF. In these patients, dobutamine stress echocardiography is used and the aortic valve is considered truly stenotic if the maximum jet velocity rises over 4 m/s with dobutamine-induced increase in stroke volume, whereas the AVA remains less than 1.0 cm^2^ [9].

IRB approval was obtained for this study.

### Non-contrast MRI

Non-contrast MRI is a noninvasive and radiation free diagnostic test that can provide critical pre-TAVI planning information and, thus, replace the use of pre-procedure invasive angiography or CTA. Advantages of MR include the ability to provide both anatomic (annular diameters, perimeter, area) measurements and quantify aortic valve stenosis (flow volumes, peak velocities) with high accuracy [10]. Recent studies have demonstrated that the information gained from both MRI and ECG-gated CTA in the pre-operative assessment of patients undergoing TAVI is reproducible [3, 7, 11–13]. MRI provides numerically similar measurements in terms of annulus size, left ventricular outflow tract (LVOT), and aortic valve area when compared to TTE [11]. MRI can also provide anatomic assessment of the aortic valve for identifying congenital valvular abnormalities, visualization of cardiac structure & function, and identification of atherosclerotic plaque, aneurysm, or dissection in the ascending aorta.

### Emerging indications for MRI

As the number of TAVI procedures increases, there will also be an increase in the number of patients who cannot be optimally evaluated with CTA, echocardiography, or stress echocardiography. Thus, non-contrast MR may have an important role in preoperative evaluation in the following groups of patients:

1. Patients with history of severe allergic type reaction to intravenous iodinated contrast medium who cannot be administered contrast medium for CTA.
2. Impaired renal function (acute kidney injury or chronic kidney injury with serum creatinine > 2 mg/dL, or GFR < 30 mL/min/m^2^).
3. Evaluation of severity of aortic stenosis in patients with poor acoustic window and low cardiac output/low gradient AS (aortic stenosis) with reduced left ventricular ejection fraction (LVEF).
4. Evaluating severity of aortic stenosis in patients with moderate stenosis by echocardiography but symptomatic for severe stenosis and who have contraindications for stress echocardiography (Appendix A) [14].

Post-gadolinium delayed enhanced imaging has been used to assess the severity of coronary artery disease in patients undergoing TAVI, and it has been demonstrated that there is decreased LVEF recovery after TAVI in the presence of significant delayed enhancement prior to TAVI [15]. Use of Gadolinium however is contraindicated in patients with poor renal function. Gadolinium enhanced MRI is not necessary for the preoperative measurements for TAVI.

### Advantages of MRI

Advantages of MRI as compared to CT or catheter angiography include: noninvasive and radiation-free imaging modality, anatomic assessment of the aortic valve for identifying congenital valvular abnormalities, performing planimetry to calculate aortic valve area, visualization of cardiac structures, characterization of the ventricular mass and function, identifying atherosclerotic plaque, aneurysm, or dissection in the ascending aorta. The type and number of available transcatheter aortic valve have evolved and increased over time. Identifying the optimal device and appropriate approach to implant depends on a number of critical measurements.

Although there are publications reviewing the usefulness of MRI, both before and after implantation, for the evaluation of TAVI, these have mostly focused on only a few aspects of the increasingly complex and comprehensive assessment needed for complete assessment prior to TAVI. This article is based on our experience of patients who underwent both contrast enhanced ECG-gated CTA and non-contrast MRI for preoperative assessment for TAVI. It provides a comprehensive review of the key measurements for the pre TAVI assessment as reported on CTA (Tables 1 and 2), most useful acquisition sequences on a 1.5 T MR scanner (Table 3), and the imaging parameters (Table 4) used for these sequences at our institution.

**Table 1:** Important aortic annular measurements, which identify the optimal Edwards Sapien transcatheter heart valve based on CTA [38] and echocardiography (http://www.edwards.com/eu/products/transcathetervalves/Pages/sapien3.aspx).

Valve	Native Annular size TEE: Diameter/Area (mm/mm2)	Mean Aortic Annulus Diameter (mm)	Aortic Annulus Perimeter	Aortic Annulus Area (mm2)	Area derived Diameter
Sapien 23 mm	18-22/338-430	19-22	60-69	300-380	20.7 – 23.4
Sapien 26 mm	21-25/430-546	23-25	72-78.5	415-490	23.4 – 26.4
Sapien 29 mm	24-28/540-683	26-28	81.5-88	530-620	26.2 – 29.5
Sapien3: 20 mm	16-19			273-345	18.6-21
Sapien3: 23 mm	18-22			338-430	20.7-23.4
Sapien3: 26 mm	21-25			430-546	23.4-26.4
Sapien3: 29 mm	24-28			540-683	26.2-29.5

**Table 2:** Important aortic measurement, which identify the optimal CoreValve trans catheter heart valve [38] (http://www.accessdata.fda.gov/cdrh_docs/pdf13/P130021c.pdf)

Valve	Aortic Annulus Diameter (mm)	Aortic Annulus Area (mm2)	Aortic Annulus Perimeter	Ascending Aortic Diameter	Sinus of valsalva: Height/Width (mm)
CoreValve 23	18–20	254.5–314.2	56.5–62.8	≤34 mm	≥15/≥25
CoreValve 26	20–23	314.2–415.5	62.8–72.3	≤40 mm	≥15/≥27
CoreValve 29	23–26	415.5–572.6	72.3–84.8	≤43 mm	≥15/≥29
CoreValve 31	26–29	530.9–660.5	81.7–91.1	≤43 mm	≥15/≥29

**Table 3:** Key MR sequence and their utility in pre-operative evaluation for TAVI

Sequence	Purpose
Three-plane localizer	Localize aortic valve plane
Axial SSFP non-ECG gated without contrast	Identify potential ascending aorta and subclavian access sites, determine size, calcification, and presence of aneurysmal dilatation of aorta
Breath held/free breathing 2D ECG-gated SSFP: Coronal Aorta, LVOT and Aortic Root	Evaluate aortic annulus, aortic valve structure, and sinus height Planimetry valve orifice area
SSFP ECG gated images: short axis stack	Calculate ejection fraction, ventricular volumes and mass
Breath held/free breathing phase contrast at aortic orifice	Calculate blood flow velocity, pressure gradient and flow volume across the aortic valve Calculate aortic regurgitant volume
3-D Navigator assisted SSFP	Coronary ostia height Aortic diameter
T2 Black Blood	Useful in presence of susceptibility artifacts from sternal wires or prosthetic valves

**Table 4:** Parameters of the key MRI sequences on a 1.5 T MRI used at our institution

Sequence	Flip Angle	TE/TR	Slice Thickness/Gap (mm)
Axial SSFP non-gated without contrast	45	1.4/3.4	6/0
Breath held/free breathing 2D ECG-gated SSFP: Coronal Aorta, LVOT and Aortic Root	45	1.4/3.4	5/0
T2 Black Blood	90	41/1791	8/0
Breath held/free breathing phase contrast at aortic orifice	25	2.7/5.6	8
3D Navigator assisted SSFP	75	1.8/4	2

#### Limitations of MRI

There are important limitations of MRI in preoperative assessment for TAVI including multiple breath holds, longer scan time, and other contraindications as discussed in appendix B. How well MRI assesses calcified plaques and porcelain aorta is not known. At present, semi-automatic analysis software is available for CT, but none is available for MRI. Not all patients can undergo MRI, and there are certain absolute and relative contraindications for MRI that the TAVI team (clinicians and cardiac imagers) should also be aware of (Appendix B).

### Aortic annulus

**Fig. 1:**
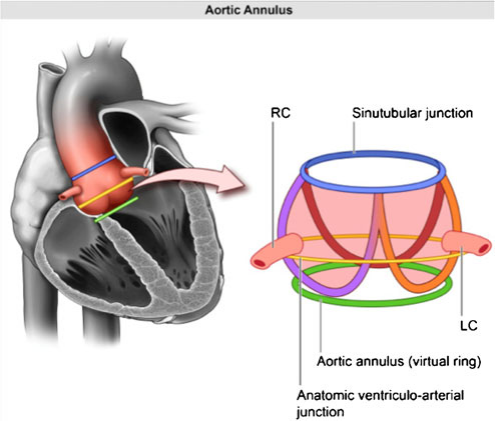
Pictorial depiction of the aortic root complex demonstrating the location of the annulus, aortoannular, ventriculoarterial and sinotubular junction

**Fig. 2:**
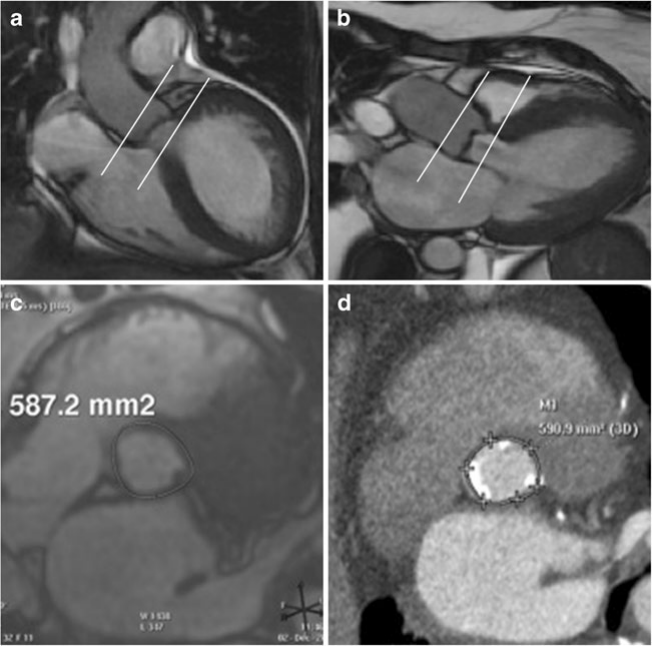
Acquisition on MRI for aortic annular plane to measure diameters, area, and perimeter. Aortic root cine stack is prescribed from coronal aorta (**a**) and left ventricle outflow tract views (**b**). Systolic image where the luminal diameter is widest, in a location just below the insertion of the valve leaflets (**c**) is identified as the annular slice. Corresponding annular image from the same patient obtained from ECG-gated CTA demonstrates similar measurement of annular area (**d**)

**Fig. 3:**
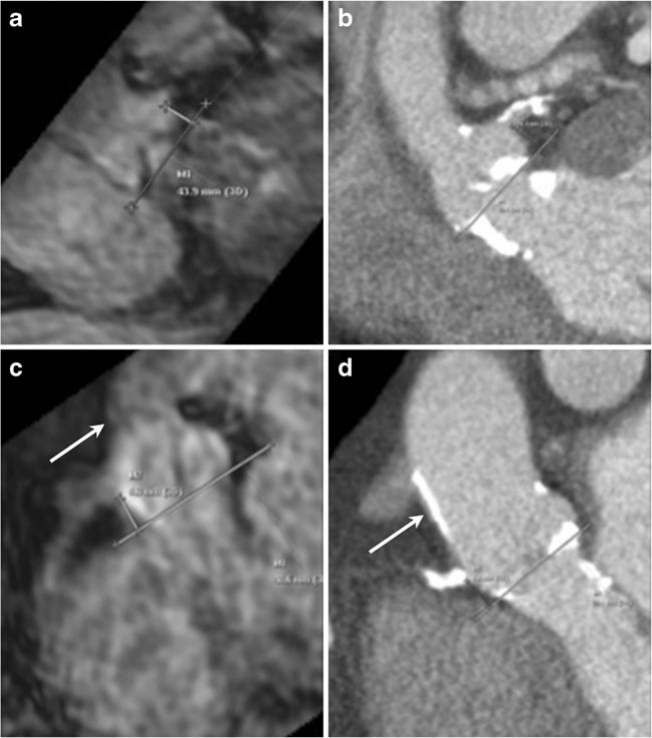
Assessment of coronary ostial height on MRI. Navigator-assisted 3-D SSFP stack of the aortic root in the late diastolic phase is acquired. Ostial height is measured from the aortic valve annular plane (**a**: left coronary artery, **c** right coronary artery). In the same patient corresponding CT images (**b**: left coronary artery, **d** right coronary artery). This MRI sequence can be also be used to assess sinus of valsalva height and width. Note the dark appearance of the anterior aortic wall and the left ventricle outflow tract due to extensive calcifications, easily seen on the corresponding CTA images

The aortic annulus is a virtual ring formed by connecting the basal hinge points of the aortic valve leaflets. The annulus is not a distinct histological entity or anatomical boundary, but represents the smallest diameter in the blood path between the left ventricle and aorta and represents the position for the implanted prosthetic valve [16] (Fig. 1). Bright blood (SSFP = steady state free precession) aortic root cine stack of 8–10 slices from diastole to systole is acquired parallel to the valve plane (Movie 1). This plane can be prescribed by planning the acquisition perpendicular to the direction of flow in aortic sinus on these two planes: coronal aorta and left ventricle outflow tract (Fig. 2a, b, respectively). This plane is similar to the suggested plane for performing planimetric assessment of the aortic orifice [11, 13, 17] as well as for performing 2-D phase contrast MRI measurements of aortic flow. Obtaining such a plane ensures that annular area and diameter measurements are obtained perpendicular to the axis of blood flow in LVOT and aorta as has been described for aortic measurements. As there is systolic descent of the annular plane, this acquisition should always begin in the left ventricle outflow tract. The annular plane is identified by the end systolic image below the insertion of leaflets by scrolling through this cine stack (Fig. 2c). This slice is used for assessing the minor & major diameters, area, and perimeter of the annulus. Annular diameters can also be obtained from a navigator-assisted free breathing diastolic phase 3-D SSFP sequence [3]. Non-contrast 3-D-FLASH MRA has been proposed to obtain diastolic annular dimensions, and using a correction factor based on systolic measurements from CTA in the same patients, a systolic corrected annular dimension can be obtained [18]. Measurement of the annulus is important for correct selection of prosthesis size, type, and to avoid damage of the annulus if the valve is oversized and avoid paravalvular regurgitation if the valve is undersized. Measurements including and excluding calcification are provided. Accurate measurement of heavily calcified annulus remains challenging, and the best method to measure such an annulus has not been determined. In our practice, on both CTA and MRI, heavily calcified annulus area and diameters are measured by including and excluding the calcification. In one study, using an in vivo model of calcium containing rings, MRI measurements have been demonstrated to be the most accurate for assessing the actual dimensions compared to CT or 3-D echocardiography [19]. When there is still uncertainty, measurements obtained by CTA/MRI are compared to annular sizing by calibrated balloon aortic valvuloplasty [20, 21]. The degree of annular calcification can also be assessed with MRI; however, how well this assessment is compared to CT is not known. In our experience, a change in signal intensity of the aortic wall should be sought as the presence of calcification leads to a darker interface on SSFP sequences (Fig. 3).

Movie 1(MOV 1458 kb)

Protruding annular calcifications > 4 mm and adherent calcification >6 mm (particularly left-sided) or calcifications with an Agatston score of > 3000 [22] are important predictors of paravalvular leaks after TAVI [23]. Comparative CT and MRI studies of heavily calcified annulus and aortic plaques have not been done. In our experience, on MRI, measurement should be obtained from the outer edge to the outer edge. In case of suspected porcelain aorta, a non-contrast CT should be obtained to better characterize the degree and extent of calcification. Evaluation of this aortic root cine stack also helps identify nodular thickening in the valve leaflets, annulus, LVOT, and for the presence of aortomitral continuity calcification/thickening (Fig. 2).

### Coronary ostia

The distance from the annulus to the coronary ostia is of importance to prevent occlusion of the coronary arteries by the displacement of the native aortic valve leaflets by the prosthesis. Risk of coronary ostia occlusion is less with the CoreValve prosthesis than with the Edwards Sapien prosthesis. For the latter, minimum distance values of 10–14 mm between the coronary ostia and leaflet insertion are usually suggested. A 3-D SSFP free breathing stack obtained in late diastole with a respiratory navigator can be used to assess the height of coronary ostia from the annular plane (Fig. 3a-b, c-d: comparative images from CTA in same patient).

### Aortic valve

**Fig. 4:**
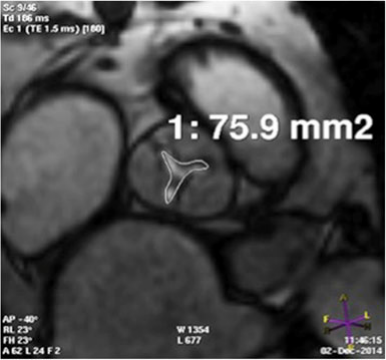
Assessment of aortic stenosis by planimetry. 2-D cine SSFP acquisition parallel to the valve plane demonstrates the narrowest opening of the aortic orifice. CT of the same patient also demonstrated similar orifice area

**Fig. 5:**
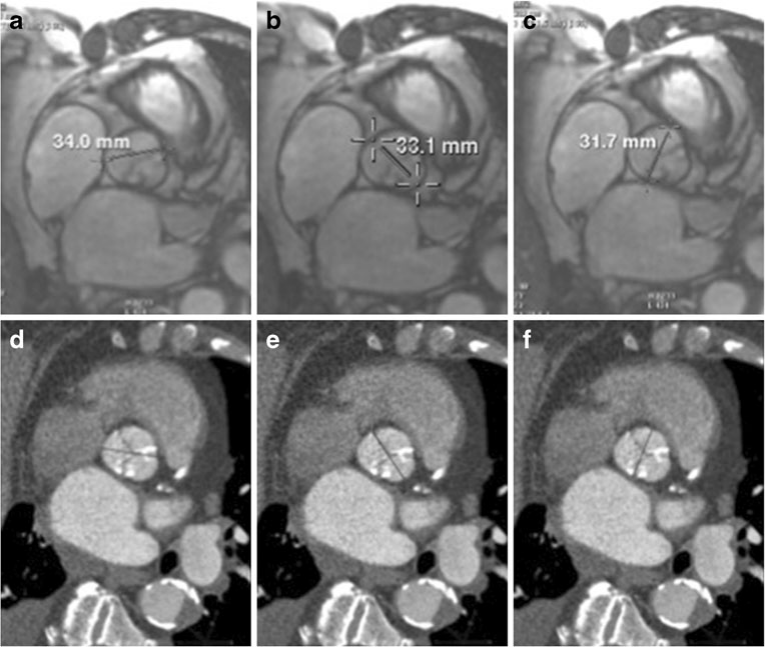
Diameters of sinuses of valsalva measured on MRI. 2-D SSFP of the aortic root in late diastolic phase is identified from the aortic root stack. Diameters are measured in mid-sinus above the aortic valve (**a**: noncoronary, **b**: left and **c** right). In the same patient corresponding CT images (**d**: noncoronary, **e**: left coronary and **f**: right coronary). Navigator-assisted 3-D SSFP or T2 black blood sequences can also be used for these measurements

Detailed anatomic assessment of the aortic valve can distinguish tricuspid vs. bicuspid, or the quadricuspid valve. TAVI has been successfully performed in selected high-risk patients with severe bicuspid aortic valve stenosis [24]. The severity of aortic stenosis is mostly quantified by transthoracic or transesophageal echocardiogram. In patients with low flow and low gradient but clinically suspected severe stenosis, dobutamine stress TTE is used to augment flow and unmask severe stenosis. Stress TTE may be contraindicated in some patients (Appendix A). Aortic stenosis can be quantified on MRI by different methods. Planimetry of maximal visible valve opening in systole on MRI (Fig. 4) correlate well with planimetry measurements obtained on CT [7, 11, 17]. Severe aortic stenosis is suggested when the aortic valve area is <1 cm^2^. The valve leaflets can be evaluated on the 2-D ECG-gated SSFP images (Fig. 5). Stenosis calculation based on the Hakki formula (aortic valve area = cardiac flow/√maximum gradient) correlate well with measurements obtained from echocardiography [25]. In addition, the velocity ratio based on peak velocity in the LVOT and aortic sinuses can also be calculated from two separate 2-D phase contrast MR acquisitions. A velocity ratio of < 0.25 is compatible with severe stenosis.

### Aortic sinus

Measurements of the sinus of valsalva diameters and height are critical for proper positioning of the device to ensure that there is no infringement of the coronary ostia (Tables 1 and 2). Sinus diameter and height measurement can be obtained from breath held or free breathing SSFP sequences (Fig. 5 a-c, e-f: comparable measurements in the same patient from CTA). Sinotubular junction height is critical for proper positioning of the device and verifying no infringement on coronary ostia especially for CoreValve. Susceptibility artefacts can limit evaluation of SSFP sequences in patients who have implantable devices or sternal wires from prior thoracic surgery. In these cases, additional non-fat suppressed T2 black blood images (coronal plane prescribed to aorta for left, and sagittal plane for right coronary artery) can be used to identify sinus diameter, height, etc.

### Aortic root

Aortic root orientation is critical for precise positioning of the device, in particular for the CoreValve. Inappropriate alignment is associated with post-procedural complications such as device malposition or distal embolization. Root angulation can be measured on coronal non-gated SSFP MRI in relation to body axis For example, if the root angle > 30°, a subclavian arterial approach cannot be used for CoreValve implantation [26].

### Ascending aorta

Assessment of the thoracic aorta for plaque burden is important. MRI is not optimal for assessment of aortic calcifications [9]. On MRI, calcifications would lead to signal voids and, hence, appear dark. Silveresa et al., studied carotid and femoral artery plaques using MRI and FDG-PETCT and demonstrated complementary values of these modalities. In this study, lipid-rich plaques were more inflamed than either calcified or collagen-rich plaques. For MRI, they measured the plaque using T2 weighted sequence [27]. Noncalcified atherosclerotic plaque burden of the thoracic aorta may increase the risk for acute renal injury post valve implantation. Aortic wall thickness that exceeded ≥ 2 mm is defined as a diseased segment [28]. Assessment of plaque thickness between echocardiography and CTA is good [29]. Noninvasive MRI also compares well with TEE for the assessment of atherosclerotic plaque thickness, extent, and composition [30]. Although there are no comparative studies evaluating CT and MRI for thickness of the aortic plaque, plaque thickness should be noted and measured using suggested comparative studies, i.e., distance between the aortic border and the point of greatest luminal protrusion [30].

In addition, evaluating the tubular ascending aorta for any aneurysm or ectasia is important for implanting a CoreValve. Heights of the Edwards Sapien prostheses are between 15–19 mm, and they do not extend beyond the aortic sinus; however, the height of the CoreValve is between 52–55 mm, and these valves extend into the tubular ascending aorta. Therefore, aortic diameter is measured for these at a distance of 40 mm from the valve plane. Significant aneurysmal dilatation is considered a contraindication for TAVI using this device [21]. All measurements of aortic diameter should be obtained perpendicular to the axis of blood flow [31].

### Transaortic access

**Fig. 6:**
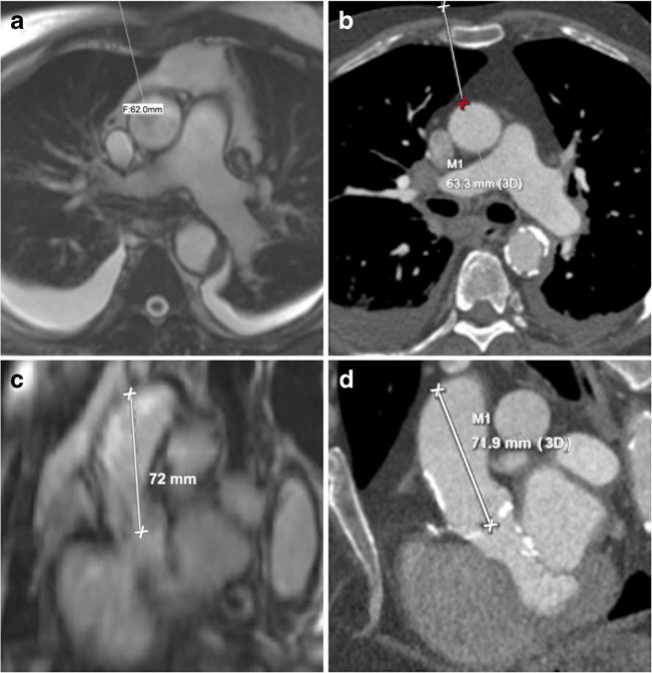
Identification of access site in tubular ascending aortic for transaortic implantation on MRI. Skin to aortic distance measured on axial non-gated SSFP sequence (**a**) similar to CTA (**b**). Access site on aorta to annular plane distance measured from multiplanar sagittal oblique reformats obtained from the same axial non-gated SSFP sequence (**c**). Same measurement in this patient obtained from CTA (**d**)

The direct transaortic approach is considered as an alternative endovascular access site in patients with unsuitable iliofemoral anatomy. Measurement of the distance from the ascending aorta access site to the skin and valve plane can be performed using axial non-gated SSFP MRI. A favourable puncture site is at the greater curve, typically the right lateral side of aorta with the following criteria: absence of calcification, atheroma, thrombi, and dissection flap or previous surgery [32]. The minimal distance from the aortic annulus to this access site needs to be at least 5 cm. This access site needs to be at least 1 cm distal to coronary venous grafts or foreign bodies (Fig. 6). The relation of the ascending aorta to the sternum helps in identifying the surgical approach. A mini J sternotomy is preferred if the ascending aorta is in midline or toward the left and >6 cm deep to the sternum. A mini right thoracotomy is preferred if the ascending aorta is right sided (>50 % of the aorta is present on the right of the sternal boarder) at the level of the second intercostal space and is <6 cm deep to the sternum [32]. How well are the calcified plaques or porcelain aorta assessed with MRI compared to CT is not known. Presence of calcification may cause loss of signal in the aortic wall (Fig. 3). When porcelain aorta is suspected, a non-contrast CT may be obtained for better evaluation.

### Transapical access

**Fig. 7:**
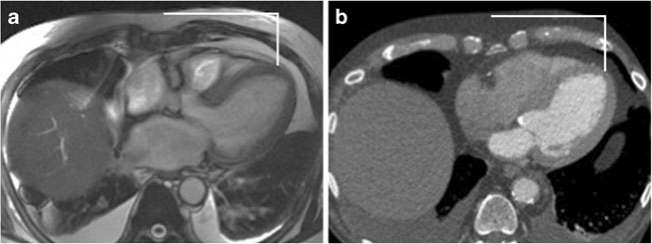
Assessment of left ventricle apex on MRI. Location of the apex in the intercostal space and the distance of this access site from the midline can be measured and marked using axial non-gated SSFP sequence (**a**). Same site location by CTA in this patient (**b**)

The transapical approach is considered as an alternative access site option for balloon expandable transcatheter valves in patients with unsuitable iliofemoral, subclavian, or aortic anatomy. The left ventricle (LV) apex is evaluated for scar/prior infarct and thrombus. Measurements of the LV apex from the sternum and skin surface can be performed using axial non-gated SSFP MRI similar to CTA (Fig. 7). Advantages of the transapical approach compared to a transfemoral approach include more reliable coaxial alignment of the transcatheter valve with the aortic annulus and greater ability to control delivery of the valve [33].

### Post TAVI paravalvular regurgitation

Aortic regurgitation (AR) is the most frequent post-procedural complication after TAVI and is linked to adverse outcomes and mortality. MRI is another quantitative imaging modality that can be used to assess aortic regurgitation after valve implantation. Transthoracic echocardiography displays higher variability and significantly underestimates AV annulus size and underestimates AR when compared to MRI [34, 35]. Compared to echocardiography, quantitative measurement of AR by MRI demonstrates better correlation with quantitative assessment by angiography [36]. MRI may be the modality of choice when there is discordance in grading AR by echocardiography [37].

## Conclusion

Pre-TAVI planning using non-contrast MRI can play a pivotal role in the assessment of the aortic root complex and thoracic access sites in patients who cannot undergo a contrast enhanced CTA either due to severe allergic reaction or severely impaired renal function. Free breathing or navigator-assisted imaging can be performed in patients with dyspnoea. MRI provides hemodynamic information with high accuracy and good correlation with echocardiography. MRI can accurately assess severity of the aortic stenosis when there is a discrepancy between clinical findings and echocardiography (because of low flow, poor acoustic window, or inability to perform a stress study). Anatomic information obtained from MRI is reproducible compared to ECG gated CTA. In the future, further studies may also establish usefulness of non-contrast MRI towards assessment of calcified plaques and peripheral vascular access.

### Electronic supplementary material

Below is the link to the electronic supplementary material.

## Appendix

A. Contraindications to stress echocardiography are: ventricular arrhythmias, recent myocardial infarction (within 3 days), unstable angina, significant left ventricular outflow obstruction, aortic dissection, and severe hypertension: systolic blood pressure > 180 diastolic blood pressure >100 mmHg or symptomatic hypertension [14].

B. Relative contraindications for MRI are: Severe claustrophobia, in which medical sedation is contraindicated or unable to resolve anxiety sufficiently, and life threatening severe arrhythmias. Absolute contraindications are: aneurysm clips, carotid artery vascular clamp, neurostimulator devices, Insulin or infusion pump, implanted drug infusion device, bone growth/fusion stimulator, Cochlear, or ear implant, and ocular foreign bodies (i.e., metal shavings).
